# Does Skipping a Meal Matter to a Butterfly's Appearance? Effects of Larval Food Stress on Wing Morphology and Color in Monarch Butterflies

**DOI:** 10.1371/journal.pone.0093492

**Published:** 2014-04-02

**Authors:** Haley Johnson, Michelle J. Solensky, Dara A. Satterfield, Andrew K. Davis

**Affiliations:** 1 Biology Department, University of Jamestown, Jamestown, North Dakota, United States of America; 2 Odum School of Ecology, University of Georgia, Athens, Georgia, United States of America; Ghent University, Belgium

## Abstract

In animals with complex life cycles, all resources needed to form adult tissues are procured at the larval stage. For butterflies, the proper development of wings involves synthesizing tissue during metamorphosis based on the raw materials obtained by larvae. Similarly, manufacture of pigment for wing scales also requires resources acquired by larvae. We conducted an experiment to test the effects of food deprivation in the larval stage on multiple measures of adult wing morphology and coloration of monarch butterflies (*Danaus plexippus*), a species in which long-distance migration makes flight efficiency critical. In a captive setting, we restricted food (milkweed) from late-stage larvae for either 24 hrs or 48 hrs, then after metamorphosis we used image analysis methods to measure forewing surface area and elongation (length/width), which are both important for migration. We also measured the brightness of orange pigment and the intensity of black on the wing. There were correlations between several wing features, including an unexpected association between wing elongation and melanism, which will require further study to fully understand. The clearest effect of food restriction was a reduction in adult wing size in the high stress group (by approximately 2%). Patterns observed for other wing traits were ambiguous: monarchs in the low stress group (but not the high) had less elongated and paler orange pigmentation. There was no effect on wing melanism. Although some patterns obtained in this study were unclear, our results concerning wing size have direct bearing on the monarch migration. We show that if milkweed is limited for monarch larvae, their wings become stunted, which could ultimately result in lower migration success.

## Introduction

In animals with biphasic life cycles, the conditions experienced by larvae can greatly affect the fitness of adults [1], [2], [3], [4]. With holometabolous insects, for example, the larval stage is the time when the organism needs to procure all of the food resources needed to form the adult tissues during metamorphosis, and any stressors or impediments they face during this stage may impair proper formation of adult morphology. For insect larvae, both the quality and quantity of food are known to be important for effective growth. The effect of host plant quality on insect fitness is a well-studied subject [5]. However, few studies have directly examined the role of larval nutrition on wing characteristics that are important for flight in butterflies, such as wing size or shape. Larval food stress produced female speckled wood butterflies (*Pararge aegeria*) with more elongated wings, but had no effect on males [6]. A study of the butterfly *Speyeria mormonia* showed that larval nutrition altered the allometric relationship between mass and wing length [7]. While this subject remains an open area of research, such results demonstrate that larval food resources can influence adult wing dimensions in butterflies.

**Figure 1:**
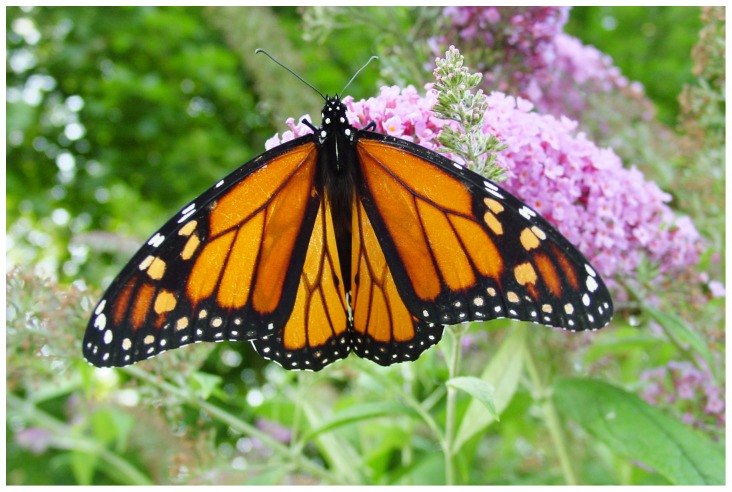
Male monarch butterfly (*Danaus plexippus*), showing the distinctive orange and black wing patterns characteristic of this species. Photograph from Wikimedia commons.

Arguably, there is no butterfly for which flight ability is more important than the monarch (*Danaus plexippus*, Fig. 1) in eastern North America. Each fall, monarchs in this population undergo a unique, long-distance migration from breeding regions in Canada and the northern United States, to a select few mountaintop sites in central Mexico [8]. There they spend the winter clustered on fir trees, and in the spring they remigrate north to produce the next generation, which will continue flying north to recolonize the breeding range [9]. For these monarchs, the success of the migration would depend largely on their flight ability, and by extension, their wing characteristics. In support of this, monarchs in migratory populations tend to have larger, more elongated wings than those in non-migratory populations [10], which demonstrates the selective force of migration on optimal wing characteristics.

Flight ability of monarchs is also tightly linked with their wing coloration. Monarch wings have distinctive orange and black patterns (Fig. 1), and the shade, or intensity of these colors varies subtly among individuals [11], [12]. Moreover, these subtle variation appear to have biological significance; monarchs with darker shades of orange (approaching red) show higher flight ability in captive settings [13], and a recent study provided evidence that the degree of black pigment is related to migration distance in wild-caught monarchs [14]. These color variations probably do not directly affect flight ability, but are likely predictors of overall condition in monarchs (and monarchs in better condition would have better flight ability). An unresolved question regarding wing color patterns in monarchs is what factors are responsible for the variation among individuals. Since the raw materials for synthesizing wing pigments (and all other adult tissues) are ultimately derived from the food acquired by larvae, the quality of the larval diet may be one important factor.

Here we report on an experiment designed to test the effect of larval food limitation (food stress) on multiple measures of adult wing morphology and color in monarch butterflies. Given the importance of wing dimensions for the migration of monarchs [10], and the known relationship between wing color and flight in this species [13], [14], we specifically focused on understanding how larval food affects wing size and shape, as well as the intensity of orange and black pigment on the wings. Larval food limitation has been shown in a variety of studies to result in reductions in adult size [15], [16], [17], [18], thus an expected outcome of this experiment was a reduction in adult wing size. We had no *a priori* expectation for how larval nutrition would impact wing shape, since this idea has rarely been tested before. Prior studies that demonstrated a reduction in various measures of adult wing pigmentation in response to poor larval nutrition [17], [19], [20] led us to predict that food stress would negatively affect wing pigmentation production of monarchs, including the shade of orange and the amount or intensity of the black pigment. Since the foodplant of monarchs (milkweed) can be limiting during the time when the migratory generation is produced (late summer/early fall), a negative effect of food stress on wing characteristics related to flight efficiency could affect the migratory success. With growing concern over large-scale losses of the monarch hostplant (milkweed) in North America [21], results of this project come at an opportune time.

## Materials and Methods

### Ethics statement

No permits were necessary for the collection of butterflies or for the experimental work presented here, and this project did not involve endangered or protected species.

### Butterflies

Before the experiment began, we first created a captive collection of adult monarchs to use as breeding stock. For this we collected monarch eggs and larvae from milkweed patches in Stutsman County, ND, from June through July, 2012. These were reared to adulthood in the lab, and upon eclosion, all adults were checked for infection by *Ophryocystis elektroscirrha* [22]. All healthy adults were placed into mesh cages containing stalks of common milkweed (*Asclepias syriaca*) for oviposition.

### Larval food experiment

We used a paintbrush to transfer first instar larvae (n = 162) within 24 hours of hatching onto a milkweed leaf, and randomly assigned each larva to one of three treatments (control, low food stress, or high food stress; n = 38, 61 and 63, respectively). We placed each larva and leaf into a plastic tub (10 cm length×10 cm width×12 cm height) with a damp paper towel on the bottom and a mesh lid for ventilation. The tubs were kept in a laboratory with natural lighting from windows. Tubs in the control treatment were cleaned and supplied with fresh leaves of cut stems of *A. syriaca* daily. Tubs in the low food stress treatment were maintained in this same way, except that larvae had no access to milkweed for 24 hours starting on the morning after they completed their molt to the 4^th^ instar. Tubs in the high food stress treatment were maintained as in the low food stress treatment, except that larvae had a 2^nd^ period of no access to milkweed for 24 hours starting on the morning after they completed their molt to the 5^th^ instar. Monarchs were reared until eclosion under these conditions, where they were frozen until measurement (below). We recorded the development time for all monarchs as the number of days from hatching to eclosion.

### Measuring wings

The right forewing was removed from each monarch specimen and scanned with a Hewlett Packard Scanjet flatbed scanner (Hewlett Packard, Palo Alto, CA). The resulting image was imported into Adobe Photoshop with FoveaPro plugins (www.reindeergraphics.com), where we obtained measurements of morphology and color following previous work [11], [13], [23], [24].We first measured the surface area of the wing and the forewing length and breadth, from which we calculated the forewing aspect ratio (length/width), which has been shown to be high in migratory populations of monarchs [10]. Next we selected the central orange cell and extracted the mean pixel hue score, which is a measure of the shade of orange, and which predicts flight endurance in monarchs [13]. This produces values ranging from 20–40 in monarchs, with lower numbers representing darker shades of orange, approaching red [12], [13]. This measure of orange color has been shown to be correlated with reflectance data from the same wing region obtained using spectrophotometers [13], [14]. We then selected all black-pigmented sections of the wing and obtained the mean pixel density value of this selection, which is an index of the shade, or darkness, of the black pigment. This number varies from 0–255, with lower values indicating darker pigment [11], [12]. Finally, we obtained the total surface area of this black pigment and calculated the percentage of forewing surface covered with black.

### Collecting migrants

Based on specific outcomes of the food experiment regarding wing morphology, we also examined wing scans from several previously-collected sets of migrant monarchs [23], [25] to evaluate whether certain patterns observed in the captive monarchs represented what would be seen in free-living monarchs. These monarchs (n = 147 total) were captured in the fall of 2005, 2008 and 2010 in Athens, GA. All were captured in the months of September, October and November, and thus were of the migratory generation of monarchs. The right forewings were scanned using the same scanner and image settings as for the lab-reared monarchs above. The same wing measurements were obtained on these specimens using image analysis (area, aspect ratio, orange hue, black density and percent black).

### Data analysis

**Figure 2:**
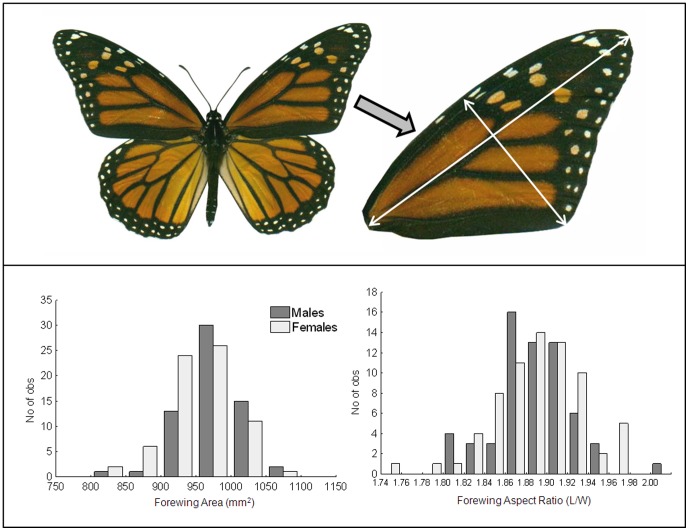
Scanned image of a monarch butterfly (top panel) with one forewing cropped to show details of forewing measurements. Lower panel shows distributions of size (area) and shape (aspect ratio) measurements in this study.

**Figure 3:**
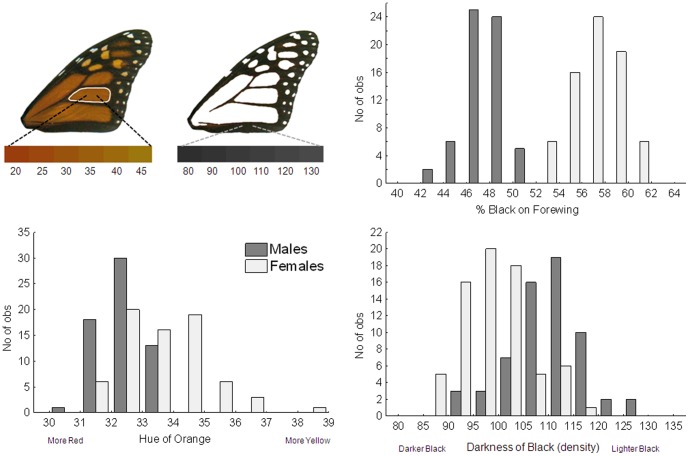
Depiction of forewing color measurements (top left), including orange hue and black density scores. Range of scores for monarchs shown beneath wings. Graphs show the distributions of all forewing color (orange hue, black proportion and density) measurements. Note that orange hue scores in monarchs can range from 20–45, but for this experiment values were between 30–40.

The response variables for the food stress experiment included monarch forewing size (area), shape (aspect ratio), orange hue, and the two measures of melanism (percent black and black density). All of these variables were normally-distributed (Figs. 2 & 3). We first determined the degree of relatedness between all response variables using Pearson correlation tests. Then, because there were correlations between several response variables (see results) we used multivariate analysis-of-variance to simultaneously examine the effect of larval food treatment (control, low and high food stress) on all response variables. Because male and female monarchs vary in wing color, sex was also included in the model, as well as an interaction term (sex*treatment). Univariate ANOVA was then used to identify specific response variables that differed among treatments and/or between sexes, and Tukey's Post-hoc tests used to determine significantly different groups. All tests were performed using the Statistica 6.1 software package [26].

## Results

A total of 139 monarchs survived to adulthood in this experiment (84% survival). Survival did not vary between treatment groups (Chi-square test, χ^2^ = 1.64, df = 2, p = 0.441). The food stress treatments prolonged the development of monarch larvae: mean development times for the control, low and high food stress treatments were 22.4d±1.1, 23.8±1.2, and 24.8±1.0, respectively. This variation was significant (one-way ANOVA, F_2,129_ = 44.08, p<0.0001), and each group was significantly different than the others based on Tukey's post-hoc tests (p<0.001 for all).

In our preliminary comparisons of wing variables, we found that several measures of adult wing morphology and color were correlated, although most of these patterns differed between male and female monarchs (Table 1). In males, larger butterflies (i.e. with larger wing area) tended to have less black pigmentation (lower % black) and their black pigment was duller (higher black density scores [low values indicate darker black]). Female monarchs did not exhibit this pattern. In both males and females, higher black proportions were associated with darker black pigment, although this pattern was only significant for females (Table 1). The only pattern observed in both sexes was an unexpected relationship between forewing shape and black density (Table 1); individuals with more elongated wings (larger aspect ratio) tended to have higher black density scores (i.e. their black pigment was lighter).

**Table 1:** Relationships between all response variables in the food stress experiment for both male and female monarchs.

Sex	Variable	Area	Aspect Ratio	Orange Hue	Black Density	% Black
**Males**	Area	1.00	-	-	-	-
	Aspect Ratio	−.0130	1.00	-	-	-
	Orange Hue	−.1055	.1174	1.00	-	-
	Black Density	.2609*	.3968**	−.0177	1.00	-
	% Black	−.2550*	.1085	.0561	−.2312	1.00

Sex	Variable	Area	Aspect Ratio	Orange Hue	Black Density	% Black
**Females**	Area	1.00	-	-	-	-
	Aspect Ratio	−.0258	1.00	-	-	-
	Orange Hue	−.2100	.1163	1.00	-	-
	Black Density	.0474	.3594*	−.1105	1.00	-
	% Black	−.0574	.0027	−.0860	−.3731*	1.00

*p<0.05,

**p<0.005.

Pearson correlation coefficients are shown for each pairwise combination. For the black density score, smaller values indicate darker pigment.

In the multivariate ANOVA examining the effects of sex and food treatment on all wing variables simultaneously, there were significant effects of sex (Wilk's λ = 0.130, F_5,122_ = 162.3, p<0.0001) and food treatment (Wilk's λ = 0.808, F_10,244_ = 2.7, p = 0.003), but not the sex*treatment interaction (Wilk's λ = 0.883, F_10,244_ = 1.6, p = 0.119). While this model showed that both gender and food treatments influenced monarch wing morphology, it does not identify which variables were affected. Univariate ANOVAs using individual wing measures as response variables showed that forewing area, aspect ratio and orange hue all varied significantly with food treatment (Table 2). Neither measure of wing melanism was affected by the food treatments.

**Table 2:** Summary of univariate results for each response variable (wing morphology traits of adult monarchs) in this study.

Response	Predictor	df	MS	F	p
**Forewing Area**	Sex	1	17713	9.36	0.0027
	Food Treatment	2	5825	3.08	0.0494
	Sex*Treatment	2	4024	2.13	0.1234
	Error	126	1891		
**Aspect Ratio**	Sex	1	0.0004	0.3	0.6121
	Food Treatment	2	0.0049	3.1	0.0474
	Sex*Treatment	2	0.0002	0.1	0.8660
	Error	126	0.0016		
**Orange Hue**	Sex	1	52.9	41.7	0.0000
	Food Treatment	2	4.2	3.3	0.0387
	Sex*Treatment	2	0.1	0.1	0.9202
	Error	126	1.3		
**Black Density**	Sex	1	3565	77.12	0.0000
	Food Treatment	2	108	2.33	0.1011
	Sex*Treatment	2	160	3.45	0.0347
	Error	126	46		
**% Black**	Sex	1	2747.2	763.79	0.0000
	Food Treatment	2	0.1	0.02	0.9783
	Sex*Treatment	2	1.9	0.52	0.5963
	Error	126	3.6		

See text for descriptions of variables. Food treatments were control (food given *ad libitum* to larval monarchs), low stress (foodplant removed for 24 hours at the 4^th^ instar) and high stress (foodplant removed for 24 hours at both the 4^th^ and 5^th^ instar).

**Figure 4:**
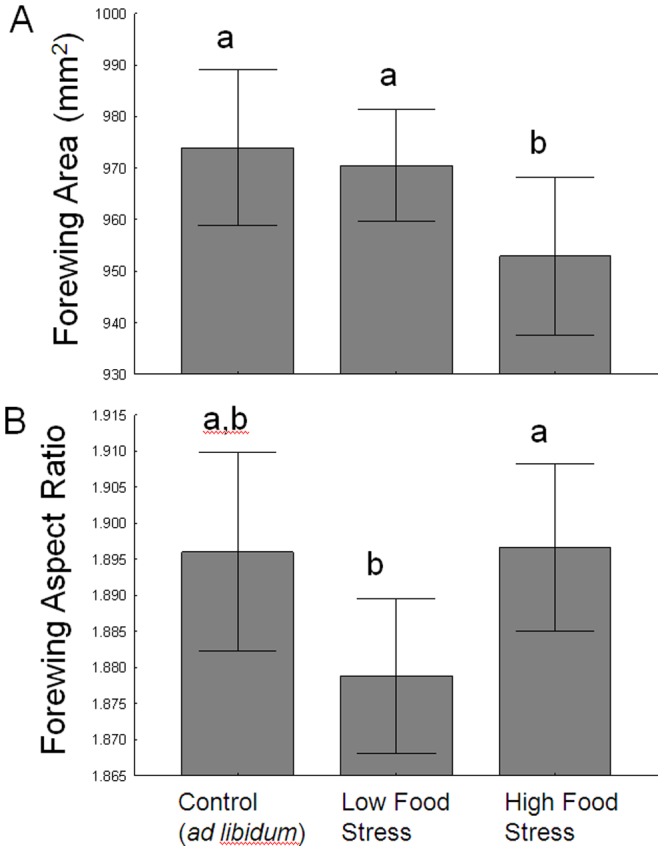
Plots showing the effect of larval food treatment on adult monarch wing size (A) and shape (B). Letters above bars indicate significantly different treatments (Tukey's Post-hoc tests). Whiskers represent 95% confidence intervals.

**Figure 5:**
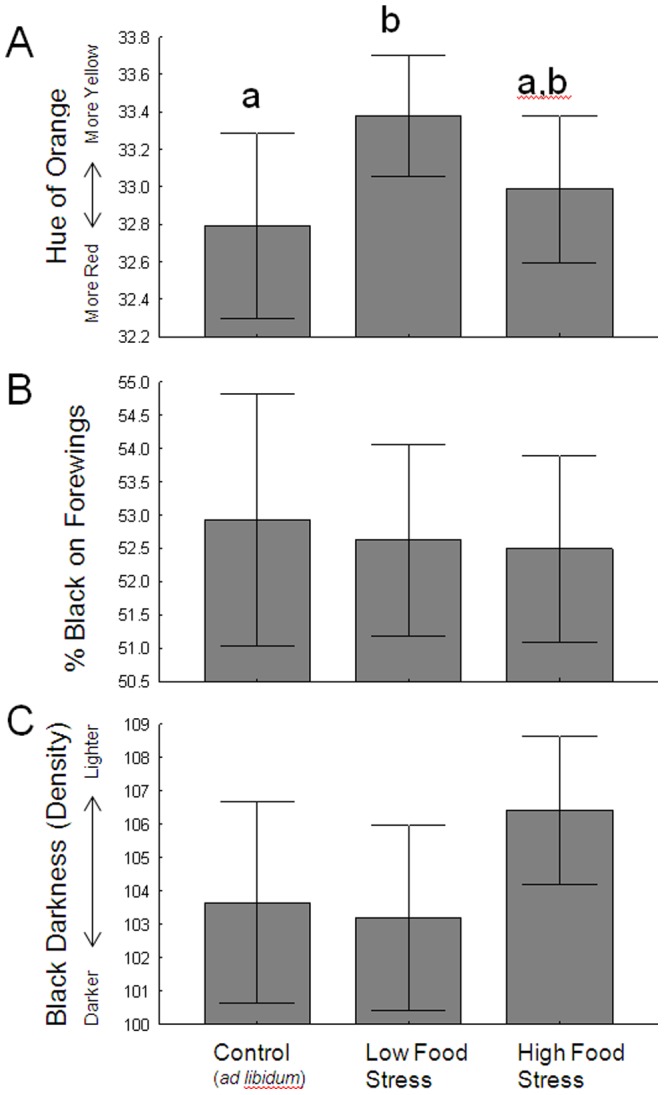
Plots showing the effect of larval food treatment on adult monarch wing color, including orange hue (A), proportion of black (B) and the intensity of black pigment (C). Letters above bars indicate significantly different treatments (Tukey's Post-hoc tests). Whiskers represent 95% confidence intervals.

Of the variables that were affected by food stress, monarch wing size showed the clearest pattern; Tukey's post-hoc tests indicated monarchs in the high food stress treatment had smaller wings than those from the other groups (Fig. 4A). To understand if this pattern was because high food stress produced ‘smaller-winged’ adults, or simply smaller adults, we separately examined how food stress affected monarch leg size (as a proxy for body size), and this investigation is presented in file S1. From that exercise we concluded that high food stress does lead to smaller butterflies overall, not just adults with smaller wings. Moreover, using leg size as a proxy for butterfly size showed monarchs from both food stress treatment were significantly smaller than the controls. The pattern observed with wing shape was ambiguous; monarchs in the low food stress treatment tended to have the least elongated wings (Fig. 4B). Of the wing color measures, monarchs in the low food stress tended to be more yellow (Fig. 5A), but there was no significant variation (due to food treatment) in the either the proportion of black (Fig. 5B) or the density of black (Fig. 5C).

**Figure 6:**
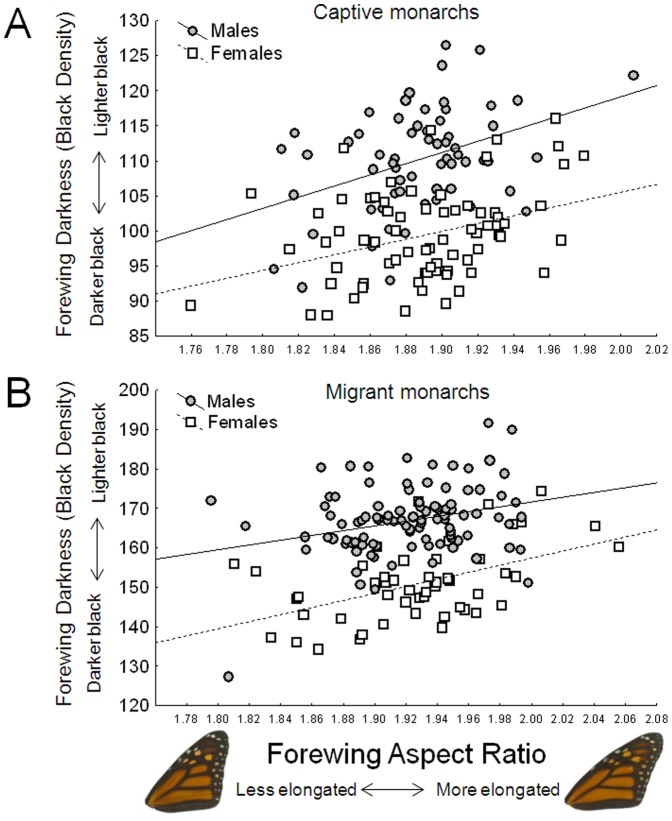
Relationship between monarch wing melanism and shape in lab-reared (captive, A) and wild specimens captured during fall migration (B). Note that the X and Y axes of both plots differ.

While tangential to the goals of the food-stress experiment, the relationship between monarch wing elongation and melanism in the captive-reared monarchs (monarchs with more elongated wings had lighter black pigmentation; Fig. 6A) was unexpected, and because of this, we examined the same variables within the wild-caught migrants. This relationship also existed in the migrant monarchs; wing elongation and black density were significantly correlated for both males (r = 0.278, p = 0.0058) and females (r = 0.496, p = 0.0003; Fig. 6B).

## Discussion

The overarching goal of this study was to determine the effects, if any, of larval food supply on adult wing morphology in monarch butterflies. We investigated this relationship by imposing two levels of food stress on captive monarch larvae: low (foodplant removed for 24 hours at the 4^th^ instar) and high (foodplant removed for 24 hours at both the 4^th^ and 5^th^ instar). Both treatments prolonged larval development time, and the high stress treatment produced smaller adults (Fig. 4A, also see File S1), which suggests that the food-deprivation regimes we imposed were stressful, and is consistent with multiple prior studies of larval Lepidoptera, including monarchs [16], [18], [27]. However, the effects of larval food restriction on other measures of wing morphology we examined were less clear.

The fact that monarchs in the low food stress treatment were still able to develop normal-sized wings (in terms of wing area, Fig. 4A), while those in the high stress treatment could not was interesting, especially given that both food stress treatments led to reductions in leg size (a proxy for body size, see File S1). This may have to do with the timing of our food restriction treatments and how they overlapped (or did not) with key developmental events in the larvae. For our low stress treatment, we restricted food for 24 hrs during the 4^th^ larval instar (but not during the 5^th^), and in the high stress treatment, food was removed during the 4^th^ and 5^th^ instars. In holometabolous insect larvae, the future wings develop from specialized internal tissue (imaginal disks) that do not begin substantially growing until the final (i.e. 5^th^) larval instar [28], [29]. Since our low stress larvae did have access to food during the entire 5^th^ instar, this may explain why their wing growth was apparently unimpeded while wings were reduced in the high stress group. Or it may simply be that one 24 hr period of food deprivation is not sufficient to compromise imaginal disk development, but two periods are.

Our comparisons of forewing shape across food stress treatments also yielded an unusual pattern, with monarchs in the low stress group tending to have the lowest aspect ratios (Fig. 4B), which indicates they had less elongated, more rounded forewings. If larval food stress compromises the development of optimal wing shape, we should have also seen a reduction in aspect ratio in the high stress group, which we did not. However, we did note that the overall means across all groups only varied by a small degree; from 1.895 to 1.878 to 1.896 for the control, low stress and high stress groups, respectively. We therefore should take care not to infer too much from these data. Moreover, such small deviations (∼0.02 between low and high) may have little biological significance in terms of flight performance for monarchs. Aspect ratios of monarch forewings usually vary from 1.6 to 2.1 (Davis, *unpub. data*, see also Fig. 6), and average ratios for migratory monarch populations tend to be above 1.93, while in non-migratory monarchs their ratios are closer to 1.88 [10]. Still, it is important to recognize that wing shape was at least partially affected by food deprivation in our experiment, so that additional study will be needed to clarify the exact mechanisms that influence development of wing shape in monarchs and other Lepidoptera.

The effects of larval food stress on adult wing pigmentation were either ambiguous (orange hue, Fig. 5A) or not significant (percent black, black density, Fig. 5B and 5C). The ambiguous pattern found with orange pigmentation in particular was not expected. Since previous studies using *Pieris rapae* and *Eurema hecabe* showed that individuals reared on low-quality diets produce paler yellow pigments [17], [30], we had predicted that food stress would also reduce pigmentation quality in monarchs. While monarchs did become paler in the low stress group (where food was restricted in the 4^th^ instar), they also should have been paler (or more so) in the high stress group (restricted during 4^th^ and 5^th^). The lack of effects of larval food supply on wing melanism, at least, is consistent with prior studies. Larval diet quality had no effect on adult wing melanism in *Malacosoma disstria* moths [31]. A previous study of monarchs showed that food deprivation for three 4-hour periods during larval development did not affect adult size or wing color [both orange hue and melanism, 32]. Moreover, in monarchs reared under high densities (leading to food limitation), there was also no effect on adult wing melanism measures [either proportion of black pigment or density of black, 33].

It is important to recognize that monarch butterflies show considerable intraspecific variation in orange brightness and the degree of black in both captive and wild settings [12], [14], [23], [24]. If food resources during larval development have little influence upon this variation, then what does? The black portions of their wings vary with rearing temperature [11], and Hanley et al. [14] reported that blackness and orange brightness were correlated in monarchs (though they were not in our study, Table 1). Environmental conditions during the larval stage could therefore play a role. However, we note that in our experiment and in many other captive studies, we often see a wide range of orange hue scores across monarchs reared individually under identical conditions of lighting and temperature [12], [24]. Given this observation, it may be that the role of lineage or family line is most important for shaping wing color of monarchs. Indeed, prior experimental work examining factors influencing the degree of black pigmentation in monarch larvae and adults found a strong effect of family line [11]. Work with other butterfly species has also demonstrated the importance of family line for determining color variation in adults [34]. Thus, offspring of high-quality parents may simply be better able to synthesize and deposit pigment onto their wings during metamorphosis.

In the course of this project we discovered an interesting relationship between monarch wing shape and pigmentation that warrants further discussion: monarchs with more elongated wings tended to have lighter black regions (Fig. 6). This pattern was present not only in our experimental monarchs, but also in prior collections of migrant monarchs, presented here for comparison. This result was unexpected, partly because a recent study of migrant monarchs found no relationship between forewing shape (elongation) and levels of wing melanism [14]. However, in that study, only the proportion of black pigment was examined (based on image analysis), not the density of black. In addition, there was no *a priori* reason to expect there to be linkages between black pigmentation and wing shape in monarchs, especially in the direction observed. If anything, given the known thermoregulatory advantages to darker wing pigment [35], [36], coupled with the monarchs' overwintering behavior in Mexico (where they cluster [and bask] on high-altitude trees), we would have expected monarchs with the *most* elongated wings [indicating higher propensity for migration, 10] to have the darkest wing pigment. In other words, those monarchs that would be most likely to reach their overwintering destination (i.e. those with the most elongated wings) should be most in need of darker wings for thermoregulation during winter. This relationship may need to be explored further with additional experiments before we can understand it fully. At the very least, the consistent findings here from the lab-reared and migrant monarchs demonstrates that information gathered from captive-reared monarchs can be helpful for interpreting patterns seen in wild monarchs.

Our experimental results have some bearing on the migration of monarchs in eastern North America. It is the last generation of larvae produced in late-summer that develop into the migratory generation, and for these monarchs, the development of optimal flight characteristics is critical. Such characteristics would include a large wing area for aiding soaring flight [37], and elongated forewings, which are common in a large number of migratory animals, including migratory populations of monarchs [10], [38], [39]. Our results show that if larval food (milkweed) were limited during late-summer, the resulting adults could still maintain their optimal wing shape, but may experience reduced size and increased development time. Similar results have been found when larval food is limited through crowding [18]. Importantly, reductions in monarch size could limit their overall migration success. In our experiment, limiting food access for 2 days during larval development led to a 2% reduction in wing area. While this difference may appear small, we note that in collections of migrating monarchs, those that are captured late in the migration season (i.e. ‘stragglers’) have significantly smaller wing sizes than the main cohort, and these differences are equivalent in magnitude to our results; late monarchs were 2% smaller (in wing length) in a study in Ontario, Canada [40] and 1% smaller in a South Carolina study [41]. In Georgia, late-migrating monarchs are approximately 3% smaller (in forewing area) than those in the early and middle part of the migration [42]. Thus, even small reductions in wing size appear to be associated with poor migration progress.

In conclusion, we found that the quality of wing pigmentation in monarch butterflies is partly influenced by larval food supply, although the effects are ambiguous and require further study. Similarly, the effect on wing shape was also not easily-interpretable. Larval food supply had a small but clear effect on wing size, but only when larvae were food-restricted at both 4^th^ and 5^th^ instars. The reduction in adult wing size after two days of larval food restriction is comparable to differences in wing size of early- versus late-migrating monarchs, which emphasizes the importance of even small differences in wing morphology for butterflies that rely heavily on flight. We also discovered an unusual relationship between wing shape and melanism, such that monarchs with more elongated wings tended to have reduced black pigmentation, and this pattern held in both captive and wild-caught monarchs. This discovery highlights the dearth of knowledge surrounding the factors that control individual variation in wing pigmentation in monarchs and other butterflies, as well as the knowledge gaps surrounding the biological implications for variation in wing pigmentation.

## Supporting Information

File S1 Summary of additional measurements (leg size) of monarch specimens, which were obtained to help interpret results concerning wing size, as well as further analyses that examined leg size variation among treatment groups.
(DOCX)
