# Supplementary material for: Does Skipping a Meal Matter to a Butterfly's Appearance? Effects of Larval Food Stress on Wing Morphology and Color in Monarch Butterflies
Source: PLoS One. 2014 Apr 2;9(4):e93492. doi: 10.1371/journal.pone.0093492 (PMC3973577; doi:10.1371/journal.pone.0093492)
Supplement: File S1 — Summary of additional measurements (leg size) of monarch specimens, which were obtained to help interpret results concerning wing size, as well as further analyses that examined leg size variation among treatment groups. (DOCX) [file pone.0093492.s001.docx]

**Does skipping a meal matter to a butterfly’s appearance? Effects of larval food stress on wing morphology and color in monarch butterflies**

**Supplemental File**

In this project, we were primarily interested in how food stress affects wing traits of monarch butterflies, and one trait we examined was forewing area, which we considered an index of butterfly size. However, because we found that food stress leads to smaller-winged individuals, we conducted the following investigation to determine if this result was because food stress led to smaller individuals overall or just butterflies with smaller wings. Using the same specimens from the original study, we measured the length of the tibia on the right front leg (not the reduced pair under the proboscis; Fig. S1), and used this as index of ‘butterfly size’. To do this we dissected the leg from the body and photographed it using a digital camera mounted to a copy stand. We used the program ImageJ (<http://rsbweb.nih.gov/ij/>) to then measure the length of the tibia (see Fig. S1).

Figure S1. Monarch butterfly with leg used for measuring indicated (red arrow). Legs were dissected from specimens and photographed (right box), and the length of the tibia was measured using image analysis software. Monarch photo taken by Pat Davis.

The tibia length data were normally-distributed (Fig. S2) and ranged from 6 to 7.5mm. Males tended to have larger tibia than females (Fig. S2).

There was a positive relationship between tibia length and forewing area (Fig. S3), although the correlation was not exceptionally strong (r=0.59), indicating the two measures of size may not be interchangeable. As a testament to this, when we examined the effect of food stress on tibia length, using the same model design as in the primary experiment (ANOVA with food treatment and sex as predictors), we found slightly different results than we did using forewing area. There was a significant effect of treatment (F_2,126_=4.80, p=0.009) but this time the monarchs were smaller in both the low and high stress groups (Fig. S4A). Comparing this graph to the plot of forewing size among treatments
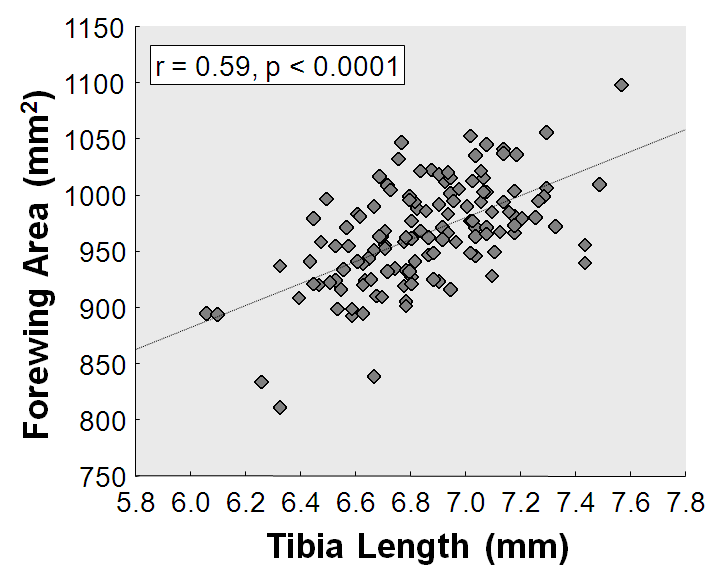
(Fig. 4A in the original study), suggests that monarchs in the low stress group grew smaller in terms of body size, but their wings did not. To further explore this idea, we extracted the residual wing area from a regression of wing area and tibia length (F_1,128_=67.6, p<0.0001), and examined how this variable was affected by food stress using the same model (ANOVA with treatment and sex as predictors). The variation in residual wing area due to treatment only approached significance (F_2,126_=2.57, p=0.080), but the two food stress treatments showed contrasting patterns (Fig. S4B).

Figure S2. Distribution of tibia length data

Figure S3. Relationship between tibia length and forewing area

Figure S4. Effect of food stress on (A) tibia length and (B) residual wing area of monarch butterflies

From this investigation we conclude that food stress leads to smaller adult monarchs (and this effect was even more pronounced than that obtained using wing size), but for reasons unknown the two levels of food stress led to differences in the ratios of wing to body size; monarchs from the low food stress treatment appeared to have larger wings for their size, while those from the high stress treatment had smaller wings relative to the body. It is possible that under mild food stress, monarchs sacrifice body size development to maintain optimal wing size for flight, but under severe food depletion, this is not possible. Another possibility (discussed in the manuscript) is that wings were not reduced in this group because the timing of food removal did not overlap with the growth of imaginal disks in larvae.
